# The Blurred Thresholds of AI-Use Disclosure: Health Professions Education Journal Editors’ Expectations of Necessity and Sufficiency

**DOI:** 10.5334/pme.2326

**Published:** 2025-12-18

**Authors:** Lorelei Lingard, Erik Driessen, Kevin Oswald

**Affiliations:** 1 Centre for Education Research & Innovation, Schulich School of Medicine & Dentistry, Western University, London, Canada; 2 School of Health Professions Education, Maastricht University, Maastricht, Netherlands.; 3 Western University, London, Canada.

## Abstract

**Introduction:**

Generative AI is a powerful resource for health professions education (HPE) researchers publishing their work. However, questions remain about its use and guidance about disclosure is inconsistent. This study explores journal editors’ experiences and expectations of AI-use disclosure, to assist journals to clarify expectations and authors to satisfy them.

**Methods:**

In this descriptive qualitative study, editors were interviewed between January 6, 2025, and May 7, 2025 using Zoom. Eligible participants were identified through journal webpages and snowball sampling. A purposive sampling strategy prioritized HPE journals and included a limited sample of general medical journals to explore transferability. Data collection and thematic analysis proceeded iteratively.

**Results:**

Eighteen participants, including 9 chief editors and 9 associate/deputy editors were interviewed. Fourteen worked in HPE journals, four in general medical journals. The analysis revealed 4 themes: 1) the basics of disclosure, made up of content expectations and process knowledge; 2) the necessity threshold, regarding which circumstances require disclosure; 3) the sufficiency threshold, regarding how much detail to include; and 4) the factors blurring these thresholds, which included the speed of change, the co-construction of standards, and the uneasy fit of some scientific principles with the AI-use context.

**Conclusions:**

While editors shared basic disclosure expectations, these were complicated by blurred thresholds of sufficiency and necessity that may exacerbate uncertainty in the scholarly community. By attending to these thresholds and the factors blurring them, and by working to articulate shared disclosure standards, HPE journals can help authors safely navigate the shifting norms of AI-use disclosure.

## Introduction

Generative AI, a type of artificial intelligence that learns from large amounts of data and can be prompted to create new content, offers a powerful resource for health professions education (HPE) researchers publishing their work [1,2,3]. However, questions remain about appropriate use [4] and published guidance is inconsistent [5]. This situation is confusing and potentially perilous: researchers risk their reputations by disclosing inappropriately.

Early questions about whether AI is being used to help write manuscripts [6,7,8] are already redundant; surveys [9] and database analyses [10,11] demonstrate that this is emerging practice. Publishers have declared that AI cannot be an author; however, beyond that declaration, debate persists regarding effective [12] and acceptable [9] uses, and guidelines often lack specifics [13]. Add a growing concern about undisclosed AI use [14,15] in a fast-changing landscape, and it is evident that researchers need more clarity [9].

Although AI-use disclosure is required for authors in and outside of health professions education (HPE) [16,17,18], guidance varies. It also evolves over time [5,19,20], evidenced by the ICJME’s shift from general recommendations to specific guidance about what and where to disclose [21,22]. Some general medical journals have shifted from discouraging AI content in 2023 [23] to anticipating it in 2024 [24]. Such evolution makes sense as AI technology advances. However, it can also create confusion. And while health professions education journals have followed ICJME’s evolution, requiring human oversight of AI outputs [25] and transparency within the manuscript about AI use [26], their policies tend to be brief: e.g., “Any use of GenAI beyond basic manuscript refinement (e.g. copyediting, formatting) must be explicitly declared, ideally in the Methods section of the manuscript [27].” Largely left to judge for themselves what constitutes ‘beyond manuscript refinement’, researchers disagree [28,29] and can’t find answers to straightforward questions: which uses should be disclosed? [30,31,27], what details are required? [32], and how will it impact peer review? [33]

Editors have a central role to play as the HPE community grapples with these questions. As leaders in the scientific community, they have a unique ability to shape the development of disclosure standards as they guide and oversee their own journal practices. However, journal editors’ perceptions and experiences are missing from the literature [5]. Therefore, to refine our understanding of current AI-use disclosure practices in HPE journals, this study asks: *What are HPE journal editors’ expectations and experiences of AI-use disclosure statements in submitted research manuscripts*?

## Methods

We used descriptive qualitative methodology [34] situated within an interpretivist research paradigm, and followed the SRQR conventions for reporting qualitative research [35].

We purposefully sampled for experienced editors of HPE journals, informed by the Medical Education Journal List 24 (MEJ-24) [36]. Because HPE journals are situated within the medical field, we also sampled in a limited way from two major families of general medical research journals to explore transferability of the work. Eligible participants were identified through journal webpages and snowball sampling. We determined thematic sufficiency [37] of the sample with reference to the principle of information power which we sought to maximize through targeted sampling of rich informants and techniques to enhance interview quality [38]. Participants were invited via email and we prioritized editorial experience in our recruitment.

We conducted individual, semi-structured Zoom interviews lasting 30–50 minutes. Interviews took place between January 6, 2025, and May 7, 2025. Questions asked editors’ expectations regarding disclosures and their journals’ experience of handling them (Supplement 1). Most participants brought anonymized examples of submitted AI-use disclosure statements to discuss.

Data collection and analysis proceeded iteratively. Using thematic analysis procedures [39,40], all authors read the first five transcripts and discussed preliminary codes. LL and KO independently identified inductive codes for the next eight transcripts, which were discussed by all authors for consistency, with attention to discrepancies requiring exploration in later interviews. KO re-analyzed all transcripts using the final coding structure, manually extracting all passages relevant to each code. LL and ED reviewed this coding and identified themes. KO re-analyzed all transcripts for themes, preparing analytic memos for discussion. Trustworthiness was enhanced through an audit trail and researcher reflexivity [41].

The authors are experienced scientific editors and authors (ED, LL) in HPE and a graduate student (KO) in information and media studies. Their experiences and assumptions about AI-use disclosure were explicitly discussed and managed through techniques such as non-leading interview questions and discrepant case analysis.

This study received institutional ethics approval from Western University’s Non-Medical Research Ethics Board (ID#125269).

## Results

Eighteen participants were interviewed (Table 1); three declined. Nine of the MEJ-24 journals were included, and four general medical journals from two prominent medical journal families. For five journals, we interviewed both the Editor-in-Chief and an associate editor they identified as particularly experienced with AI disclosures. At our request, fourteen participants brought anonymized examples to discuss. All general medical journal editors and all but one HPE editor reported seeing disclosures in submitted manuscripts in the preceding months. Overall, HPE editors conveyed more limited experience with disclosures than general medical editors.

**Table 1:** Participant Demographics.


DEMOGRAPHIC	# (%) PARTICIPANTS N = 18

**Journal type**	

Health professions education journal	14 (77)

General medical journal	4 (22)

**Role**	

Editor in Chief	9 (50)

Deputy/Associate editor	9 (50)

**Participant Country**	

Australia	1 (5)

Canada	4 (22)

Netherlands	1 (5)

Oman	1 (5)

Singapore	1 (5)

UK	3 (5)

USA	7 (38)

**Gender**	

Female	10 (55)

Male	8 (44)

**Years of editor experience**	

<5	1 (5)

>10	17 (94)


Four themes were identified across the dataset: 1) the basics of disclosure, 2) the necessity threshold, 3) the sufficiency threshold and 4) blurred thresholds.

### The basics of disclosure

Participants shared a sense of the basic content they expected in an AI-use disclosure. Most expected authors to name the tool, specify the task it was used for, and include a statement attesting responsibility for the material: “We ideally would look for a statement that describes, well, that confirms whether or not AI has been used. What type of AI has been used and the reason why it’s been used. And we also would like the authors to consider adding a statement into the manuscript regarding this…” (P4). They noted positive features in disclosures they brought to discuss:

This author acknowledges that “specific generative AI tools have been used during the writing of this article, was used to suggest paraphrasing of author-written text, and to provide ideas for the structure of the introduction and conclusion sections. Not all of these suggestions or ideas were used, and all AI-generated content was reviewed and adapted by the author prior to submission.” So, now, that’s a nice one. (P17)

In editors’ experience, however, these basics were often missing or mis-placed. Disclosures were “a little messy” (P12) and it wasn’t always clear that authors were aware of requirements: “The policy says we suggest you put it in the methods or some other logical place… I don’t think I’ve seen it in the methods section yet. I’ve seen people just put at the end, like in amongst … acknowledgements, disclosures, stuff like that.” (P14).

While almost all participants talked with confidence about basic content expectations, they were more tentative in describing disclosure processes in their journals. Nearly half did not know how AI-use disclosure was prompted in their journal’s online submission architecture: one admitted “I need to check, I don’t know, I think I’ve always assumed okay… I really don’t know” (P16), while others commented that they had no visibility into technical changes in the submission system affecting AI disclosure and would need to do a faux manuscript submission to see them. Many participants reported an online Yes/No checkbox prompt for AI-use with an open text box, while one acknowledged that online architecture was “a moving target” at their journal. (P6) Participants varied in their sense of whether AI-use disclosures were visible to peer reviewers, but most believed that not to be the case: “I’m trying to think if they would see it. Uh, I don’t think they would… I don’t think they would see an AI statement.” (P5)

As the interview continued, participants complicated their initial description of the “basics” of disclosure content with two nuances: when is disclosure necessary and what level of detail is sufficient?

### The necessity threshold

The ‘necessity threshold’ theme captured participants’ discussions about whether, and when, AI-use necessitated disclosure. This was not unambiguous: as one editor acknowledge, “What counts as GenAI has also been something I’ve wondered.” (P8) Using AI for spelling or grammar was not perceived to warrant disclosure because such tools have “been around forever`” (P10), but editors also acknowledged that “that pushes it back to the author to say, well, what’s AI?” (P2). All editors perceived that substantial use must be disclosed, but they defined this variably. Some put the threshold at “intellectual” (P4) work, but the distinction between superficial and substantive use was acknowledged to be “a very gray line” (P8). Editors reflected on the implications of a lack of clarity regarding the necessity of disclosure: “Okay where’s the threshold? I wonder as a scholar, if people just don’t know. That’s one of the reasons that they’re not disclosing, because they’re just not really sure what’s okay and what’s going to get editors and peer reviewers a bit squinty-eyed” (P8). They also acknowledged that journals may be uncertain about what uses meet the necessity threshold, particularly with regards to uses they saw disclosed infrequently. One example offer was when authors

“use AI as almost like a mock peer review kind of thing. …you know, those seem to be the types of uses that aren’t necessarily being disclosed very often. And I’m wondering, is that the kind of thing that you do want to know about, and how do you create a policy that creates safety for authors to be comfortable in doing that?” (P18)

As such comments suggest, editors recognized the question of necessity as value-laden and related to author safety and comfort. This extended to the question of sufficiency as well.

### The sufficiency threshold

The ‘sufficiency threshold’ theme captured participants’ reflections on what details AI-use disclosures should include. These reflections complicated the basic expectations stipulated early in the interview, with participants diverging on how much detail was required. Some editors wanted authors to “make sure that it’s fully transparent” (P4), while others preferred disclosure to “scale” (P8) with AI’s impact on the content:

I wouldn’t care what they put into their prompt if it just said something like, ‘I wanted a summary abstract’ … but if it was something along the lines of ‘we got the AI tool to do a first draft of our discussion’, I might get a little bit more interested into exactly what they put into that prompt. (P14)

Sufficiency was perceived to be changing over time. One participant expressed that recommendations to include prompts and chatlogs as supplementary files had declined relative to the first year post-ChatGPT, while another noted that “the disclosures we received in 2023 and 2024 were a lot more detailed than the disclosures that we have received more recently. …what I see now are very short disclosures — ‘we used AI tools to improve the grammar or flow’” (P13).

The level of detail in a disclosure was a matter of trust, according to participants. Vague disclosures raised concern, while detailed disclosures were seen to build trust: “They went point by point on exactly how they used it, what process they did to evaluate the product from the AI, their responsibility they took and the end result in terms of what went into the paper. And it was refreshing. It was wonderful. It was to say…if this could be the standard, I would be very happy with this.” (P2) However, participants recognized that “there’s a tension between, you know, we say we want you to be transparent, but if we’re transparent you’re going to punish us somehow” (P5). Editors understood that authors might limit the details in their AI-use disclosure to avoid scrutiny: “who’s going to be the one to step up and be that guinea pig, you know, somebody that’s willing to have those conversations without being redlined or not published in these journals. Right. That’s really hard to do” (P1). In summary, most participants acknowledged that more detail was not always better: details may either build trust or undermine it.

### Blurred thresholds

Participants’ perceptions and experiences suggested that the thresholds of disclosure necessity and sufficiency were not straightforward. They discussed three factors that blurred these thresholds: the speed of change, the philosophy of co-construction, and the uneasy fit of some scientific principles.

Participants reasoned that journal policies remained vague because the speed of change would make any policy outdated: one described the situation as “a very fast-moving space… that we’re going to have to constantly revisit” (P16). HPE editors in particular reflected that “we’re early days… six months ago, sort of we didn’t have a policy like a lot of places” (P7); and there was a perception that “the whole field has more questions than answers” (P1). Some participants described their editorial experiences as limited: “it’s very rare still for me to see these disclosures” (P6). And there was a perception that HPE editors “only know philosophically what they think a good one should look like, because they actually haven’t seen enough of them” (P9).

Participants characterized the scientific field as co-constructing an understanding of AI-use disclosure, as authors submit their AI-use disclosures and journals/editors judge whether they fulfill their purposes. As one editor explained, “When I say co-construction, what I really respect in the … policy of ‘tell us how you use the AI’ is that we editors start to look at ‘yeah that’s a good idea or that’s not a good idea’. This is the thing. So that we all learn together in terms of the way you should use it. And so, it’s … an evenhanded, non-judgmental way of surfacing issues.” (P11) However, participants recognized that co-construction creates uncertainty. Some mentioned the power differential in publishing, recognizing that disclosure statements are viewed as how we “police this situation” (P5) and authors are “worried that [disclosure’s] going to negatively impact their article” (P7). All editors explicitly noted their journal’s non-punitive stance: “the intention is not to punish authors” but to “develop our thinking and or moralities around this topic” (P1). However, there was also a sense that “it’s a bit of a liminal space… in between two worlds we are now. We are also a little bit lost, I think” (P16). Editors also recognized that co-construction of disclosure standards could be complicated by inconsistencies between policy and practice: “It says right in there: ‘checking this box will not affect the decision about your article’. That being said, I’ve seen conversations in the background where people said,’ oh, they used AI, we need to discuss this’. So, I think we’re talking out of both sides of our mouth” (P7).

Disclosure thresholds were also complicated by the application of incompatible scientific principles, particularly reproducibility and transparency. Some participants advocated disclosure to ensure research would “be reproducible” (P12), and wanted sufficient information “to interpret, to appraise, to possibly replicate the work” (P6). One editor characterized this as the “bar” that a good AI-use disclosure should meet: “The standard or the intention would be replicability. Right? And I don’t think we’ve seen many papers at all where there’s disclosure to that level. You asked … what is the bar. … And I think that’s the bar.” (P12) However, others explicitly dismissed reproducibility as incompatible with AI: “you can put the same prompt into it twice in a row and get different response – there’s nothing reproducible about your interactions with GenAI” (P11). Transparency was also recurrently invoked as a criterion for robust disclosure: “the most transparent you can be is providing the actual prompts, almost like a script of what you did. That’s like, ‘here’s what I did. Go for it. Look at it all you want.’” (P7) However, other editors questioned how transparent a disclosure could be, given the “black box” (P17) of AI and users’ poor understanding of how it produces outputs. As one put it, “We are uncomfortable with the notion that we don’t know what’s going on in the black box. And it works. And we hate that it works and we don’t understand how the thing works.” (P11)

## Discussion

HPE journal editors occupy influential positions in our field, and they can shape how the field responds to emergent publishing issues such as the role of AI. Therefore, their perceptions and experiences should inform current conversations about what constitutes appropriate AI-use disclosure. This first study to interview HPE journal editors found a shared sense of disclosure principles: disclose tool, specify task, attest responsibility. Shared principles are a good starting point for developing community standards, and these three resonate with existing literature on disclosure. However, we found that these principles are not as straightforward as they seem. They are complicated by editors holding varying views regarding when a disclosure is necessary and how much information is required for a disclosure to be sufficient.

Understanding that disclosure thresholds are blurred, even for editors, can be empowering for authors. The necessity threshold alerts authors to the challenge of judging which circumstances require disclosure. With disclosure still relatively uncommon according to HPE participants, this threshold may be hard for authors to discern. Further, if the HPE research community understands AI is being used but rarely sees disclosure [10,39], this may encourage assumptions that disclosure is unnecessary. The sufficiency threshold flags the challenge of anticipating whether details will be positively or suspiciously received. Some editors advocated maximum detail; others preferred disclosures scaled for AI-use. And timing matters: early advice to include prompts and chatlogs [42] is changing.

We identified three factors influencing blurred thresholds. Below we make recommendations for editors and authors, taking these factors into account to clarify disclosure expectations. While we focus these implications on the field of HPE, our limited sampling of general medical journal editors suggests the likelihood of similar complexities; therefore, we encourage readers applying to general medical journals to consider the transferability of our results to those contexts.

Table 2 summarizes insights into recommendations for editors and journals, while Table 3 summarizes insights into guidance for authors. ChatGPT 4o was prompted to prepare a draft of Table 3 Column 1 based on a summary of the ‘basics’ results; no raw data was entered into the AI. We edited this draft significantly, including adding Column 2. We take responsibility for this version.

**Table 2:** AI-Use Disclosure Guidance for HPE Editors & Journals.


**Understand how your journal’s online architecture shapes AI-use disclosure**

Review online submission architecture to understand how disclosure is prompted in your system: Is refinement required given speed of change? Understand how disclosures populate from the online system into the editorial process: Who sees disclosures and where in the process?

**Provide clear, detailed guidance about what your journal expects in AI-use disclosure**

Review guidance for authors: Is it sufficient? Does it reference principles such as reproducibility in problematic ways? Provide explicit examples to show authors where your journal sets the necessity & sufficiency thresholds: Don’t over simplify – how should authors handle blurry situations? Remind authors to attest to accountability regardless of where the disclosure appears in the manuscript.

**Educate authors, editorial team and peer reviewers to cultivate safety**

Educate the editorial team to ensure the co-construction philosophy is universally understood. Make explicit to authors that disclosure is non-punitive. Provide guidance to peer reviewers regarding AI-use disclosure: What is their role?


**Table 3:** AI-Use Disclosure Guidance for HPE Authors based on Editor Expectations.


DISCLOSURE BASICS	COMPLEXITIES OF SUFFICIENCY AND NECESSITY

**State how you used AI**. Example: “We used ChatGPT 4o (May 2024 version) to shorten our abstract and strengthen our study limitations.”	**Distinguish between technical & intellectual use**. Intellectual uses are of more concern to editors and require more explanation. Example: “We used ChatGPT 4o (May 2024 version) to help brainstorm counterarguments in the Discussion. We prompted counterarguments by discipline (e.g., psychology) and research paradigm (e.g., positivism).”

**Be specific: tool, tasks, location**. Include at minimum: the tool name, version or date (if known), the tasks performed, and the manuscript section(s) affected.	**Be strategic about the level of detail**. More detail is not always better or feasible. Scale down details for technical use (e.g., editing grammar); scale up for more intellectual use (e.g., creating interview prompts). Editors may request more details as the manuscript nears acceptance.

**Add a responsibility statement**. Attest that you are accountable for the content. Example: “All AI-assisted content was reviewed and verified by the authors, who take full responsibility for its accuracy and integrity.”	**Indicate *how* you verified content**. Verification is a process, not a promise. Be transparent about how you verified. Example: “We verified accuracy of AI-produced text by conducting our own PubMed search and reviewing key sources. Where we found discrepancies or oversimplifications, we edited the AI text as needed.” Consider what aspects of AI-use might not be transparent or verifiable.

**Put the disclosure in your manuscript**. Editors prefer this in the Methods or other section as relevant, rather than in Acknowledgements.	**Aim for credibility, not necessarily reproducibility**. Share your thinking process; acknowledge inconsistencies in AI output; describe how you iterated prompts to achieve better results. If you complete a checkbox or text box during online submission, ensure coherence with manuscript disclosure text.

**When in doubt, disclose**. If the AI use shaped ideas, text structure, or scholarly content—not just grammar—editors generally expect disclosure.	**Don’t avoid disclosure in fear of punitive action**. Journals and authors are co-constructing the norms of disclosure, learning together in a fast-changing situation as AI technology and literacy develop. Disclose in this spirit. Provide detail; editors can request unnecessary details be removed upon acceptance.


The speed of change makes AI-use disclosure a fluid environment. All participants noted this fluidity; many cited it as justification for not providing more comprehensive disclosure guidance. However, a fluid environment requires more, not less, author guidance. With a ‘cottage industry’ of frameworks [43,44,42], authors need direction. Editors need it too, particularly given HPE editors’ reports of limited experience with AI-use disclosure and their uncertainty about how journal online submission architectures prompted disclosure and who has access to disclosures across the review workflow. We recommend that editorial teams analyze their own systems to clearly identify: Is disclosure prompted or not? Where do disclosures entered into the online submission form populate in the manuscript? Who sees disclosures and when in the review workflow? Furthermore, if editorial teams discover unclear or inconsistent procedures in their systems, they might use emerging resources to clarify their positions and educate editorial team members. For instance, a recent framework that distinguishes mandatory, optional, and unnecessary disclosure [5] could stimulate productive discussion and explicit setting of necessity thresholds that are contextually relevant for HPE and temporally appropriate as AI continues to evolve.

Co-construction also blurs the necessity and sufficiency thresholds. Editors expected that authors, by declaring AI-use details, would help journals gradually articulate the dimensions of appropriate AI-use and disclosure. Editors understood that co-construction required authors to take risks, although they described a non-punitive approach to insufficient or absent disclosures. Whether this non-punitive approach is understood by authors is unclear; editors should consider making more explicit reference to how this is achieved during the review process to increase authors’ sense of safety in disclosure. Co-construction might be enhanced in the HPE field – and risks mitigated – by drawing on established publishing norms. For example, journals might use the Artificial Intelligence Disclosure framework [45] that adapts the CRediT taxonomy [46] of contributor roles to AI-use to create more precise guidelines for authors. Because it taps into our field’s familiarity with the expectations for authorship contribution, this framework could prove a helpful resource while we co-construct our disclosure norms in a fluid environment.

Finally, our results suggest the need for critical discussion of how longstanding scientific principles fit with AI. While reproducibility and transparency feature prominently in our dataset and in published guidelines [43] for AI-use and reporting, the inconsistency of AI responses and the black box nature of AI raises questions about their fit. We are not suggesting that journals discard traditional scientific principles, which may be more, not less, important with the rise of AI-use [24]. However, we do advocate explicit and critical discussion of how these principles shape disclosure policies, and whether they require reframing to address incompatibilities.

This study has several limitations. Our thematic findings cut across both HPE and general medical journal editor data; however, the latter sample is insufficient to fully portray disclosure expectations in the broader medical journal context. We focused on editors’ perceptions; the anonymized examples referenced in editor interviews were neither collected nor analyzed. Work is underway to analyze published disclosures to understand content and placement patterns. Our purposive sampling of experienced and AI-familiar journal editors excludes the perspectives of more AI-cautious editors and of peer reviewers, whose expectations and experiences will also influence the emerging norms of AI-use disclosure. In spite of these limitations, and the reality that a fluid AI environment renders findings quickly outdated, our study offers timely insights into the current ambiguities surrounding AI-use disclosure in HPE.

## Conclusion

“When in doubt, disclose”. Seems simple, but blurred thresholds of necessity and sufficiency complicate AI-use disclosure. Disclosure rules need to be explicit and dynamic. Editors and journals should review how they prompt and structure AI-use disclosure, and track its influence on peer review and editorial decision-making. They should be more transparent about their non-punitive stance in order to communicate a sense of safety for authors whose disclosures enable co-construction of shared standards. Authors should treat disclosure norms as evolving and recognize that their disclosures are co-constructing these norms. Best practice in the meanwhile is to disclose with sufficient detail and transparency that readers can judge how AI influenced the work and how the authors orchestrated and evaluated that influence. By doing so, authors can protect their own credibility and integrity as they assist the field to find its way.

## Previous presentations

This article was submitted as a preprint to medRxiv on July 17, 2025.

## Additional File

The additional files for this article can be found as follows:

10.5334/pme.2326.s1 Supplementary File 1. Interview guide.
